# Sexual health as a portal to men's health: a problem turned around into an opportunity

**DOI:** 10.1111/j.1742-1241.2007.01657.x

**Published:** 2008-02-01

**Authors:** R Shabsigh, S Arver, K S Channer, I Eardley, A Fabbri, L Gooren, A Heufelder, H Jones, S Meryn, M Zitzmann

**Affiliations:** 1 Division of Urology, Maimonides Medical Center, Columbia University Brooklyn, NY, USA; 2 Center for Andrology and Sexual Medicine, Karolinska University Hospital/Huddinge Stockholm, Sweden; 3 Royal Hallamshire Hospital Sheffield, UK; 4 Department of Urology, St James University Hospital Leeds, UK; 5 Universita di Roma Tor Vergata Rome, Italy; 6 Endocrinology, Vrije Universiteit medical center Amsterdam, The Netherlands; 7 Endocrine Clinic & Research Unit Munich, Germany; 8 University of Sheffield Medical School Sheffield, UK; 9 Institute for Medical Education, University of Vienna Vienna, Austria; 10 Institute of Reproductive Medicine Münster, Germany

The prevalence of genitourinary problems and associated health concerns is high in middle-aged and older men. A representative survey among men in Australia over the age of 40 years indicated that 34% reported one or more reproductive health disorder (1). There is a belief that men, compared with women, make less use of health services. This was not confirmed in the above study. About 88% of men in this study had consulted a health professional in the past 12 months, and health service utilisation increased with age. However, the research showed also that sexual and reproductive health problems are often not explicitly discussed with a health professional. Clinicians, while often seen as the primary source of contact for reproductive health problems, are often reluctant to initiate discussions with older patients about sexual health (2). In our view many opportunities to improve ageing men's health are thus not utilised. We propose a view here that genitourinary (including sexual) health is a ‘portal’ to men's health.

Symptoms of genitourinary problems, such as erectile dysfunction (ED) and lower urinary tract symptoms (LUTS) (3) are significantly associated with the metabolic syndrome and type 2 diabetes mellitus. The main components of the metabolic syndrome are abdominal obesity, insulin resistance, hypertension and hyperlipidaemia, all of which are etiological factors of ED. The metabolic syndrome and one of its consequences, type 2 diabetes mellitus, are, in turn, associated with lower-than-normal serum testosterone (T) levels (late onset hypogonadism). It is noteworthy that late onset hypogonadism is frequently symptomatic. In men 45 years or older surveyed in 130 primary care practices in the USA, the presence of one or more symptoms occurred in 66% of men deemed hypogonadal based on their T levels(4).

The relationship between the metabolic syndrome and type 2 diabetes mellitus and hypogonadism is probably bidirectional. A large degree of obesity and hyperinsulinism suppresses production of T, but low T levels predict the metabolic syndrome and type 2 diabetes mellitus. So, there is a close relationship between ailments frequently occurring in the ageing male (ED, LUTS, visceral obesity, cardiovascular disease and diabetes mellitus) on the one hand and hypogonadism on the other. This close relationship probably indicates that late onset hypogonadism is an expression of poor health, as may be concluded from a recent study. Low testosterone levels were associated with increased mortality in male veterans (5), but this association was not confirmed in another study (6). This is as yet not sufficient evidence to treat all ageing men with hypogonadism with T. Lifestyle changes are associated with improvement in sexual function in about one-third of obese men with ED at baseline. Weight loss and increased physical activity appeared to have a favourable effect on erectile and endothelial functions in obese men (7,8). The Massachusetts Male Aging Study has estimated the frequency of ED progression and remission among ageing men, and assessed the relation of progression/remission to demographics, socioeconomic factors, comorbidities and modifiable lifestyle characteristics. Natural remission and progression occur in a substantial number of men with ED. Age and body mass index were associated with progression and remission of ED. The association of body mass index with remission and progression, and the association of smoking and health status with progression, offer potential avenues for facilitating remission and delaying progression. The benefits of such interventions for overall men's health may be far reaching and confirm the position that ED is a portal to men's health (9).

Treatment of ED when associated with hypogonadism may entail T administration (10) and these interventional studies may provide an opportunity to assess benefits, yield, and justification of T administration on the closely interrelated ailments of ED and the metabolic syndrome of which epidemiologically hypogonadism is a correlate. The first results of T administration to elderly men show that this may lead to improvement of both ED and elements of metabolic syndrome (11) but the administration of T to men with diabetes mellitus is disappointing (12).

Shabsigh (13) have argued that ED can calculate men's health risks. Elements in the calculation of health risks (hypertension, diabetes, angina or hyperlipidaemia) in men presenting with ED are: health status on a scale of 1–7 (1 = excellent, 7 = poor), waist size, severity of ED, presence/absence of a sexual partner. The calculation produces scores of ranges of 1–7. If the score is 1.5–2.5 = medium risk (30–59% probability); ≥ 2.5 = high risk (≥ 60% probability of having the condition) and < 1.5 = low risk (< 30% probability).

In conclusion, physicians confronted with elderly men with genitourinary problems are in a unique position to address general health questions of the patient. A holistic approach will not only benefit the presenting complaint but improve the general health and well-being of the patient.
